# Web-based Real-Time Case Finding for the Population Health Management of Patients With Diabetes Mellitus: A Prospective Validation of the Natural Language Processing–Based Algorithm With Statewide Electronic Medical Records

**DOI:** 10.2196/medinform.6328

**Published:** 2016-11-11

**Authors:** Le Zheng, Yue Wang, Shiying Hao, Andrew Y Shin, Bo Jin, Anh D Ngo, Medina S Jackson-Browne, Daniel J Feller, Tianyun Fu, Karena Zhang, Xin Zhou, Chunqing Zhu, Dorothy Dai, Yunxian Yu, Gang Zheng, Yu-Ming Li, Doff B McElhinney, Devore S Culver, Shaun T Alfreds, Frank Stearns, Karl G Sylvester, Eric Widen, Xuefeng Bruce Ling

**Affiliations:** ^1^ Tsinghua University Beijing China; ^2^ Stanford University Stanford, CA United States; ^3^ Zhejiang University Hangzhou China; ^4^ HBI Solutions Inc Palo Alto, CA United States; ^5^ Tianjin Key Laboratory of Cardiovascular Remodeling and Target Organ Injury Pingjin Hospital Heart Center Tianjin China; ^6^ School of Medicine Zhejiang University Hangzhou China; ^7^ HealthInfoNet Portland, ME United States

**Keywords:** electronic medical record, natural language processing, diabetes mellitus, data mining

## Abstract

**Background:**

Diabetes case finding based on structured medical records does not fully identify diabetic patients whose medical histories related to diabetes are available in the form of free text. Manual chart reviews have been used but involve high labor costs and long latency.

**Objective:**

This study developed and tested a Web-based diabetes case finding algorithm using both structured and unstructured electronic medical records (EMRs).

**Methods:**

This study was based on the health information exchange (HIE) EMR database that covers almost all health facilities in the state of Maine, United States. Using narrative clinical notes, a Web-based natural language processing (NLP) case finding algorithm was retrospectively (July 1, 2012, to June 30, 2013) developed with a random subset of HIE-associated facilities, which was then blind tested with the remaining facilities. The NLP-based algorithm was subsequently integrated into the HIE database and validated prospectively (July 1, 2013, to June 30, 2014).

**Results:**

Of the 935,891 patients in the prospective cohort, 64,168 diabetes cases were identified using diagnosis codes alone. Our NLP-based case finding algorithm prospectively found an additional 5756 uncodified cases (5756/64,168, 8.97% increase) with a positive predictive value of .90. Of the 21,720 diabetic patients identified by both methods, 6616 patients (6616/21,720, 30.46%) were identified by the NLP-based algorithm before a diabetes diagnosis was noted in the structured EMR (mean time difference = 48 days).

**Conclusions:**

The online NLP algorithm was effective in identifying uncodified diabetes cases in real time, leading to a significant improvement in diabetes case finding. The successful integration of the NLP-based case finding algorithm into the Maine HIE database indicates a strong potential for application of this novel method to achieve a more complete ascertainment of diagnoses of diabetes mellitus.

## Introduction

Diabetes mellitus (DM) is a leading cause of mortality and morbidity and accounts for significant burden of disease worldwide [1,2]. In the United States, 9.3% of the population or 29.1 million people were reported to have diabetes in 2013, plus an estimate of 8.1 million people with undiagnosed diabetes [3,4]. Diabetes is a metabolic disorder caused by a high concentration of glucose in the blood stream. If untreated, diabetic patients will eventually develop a range of complications. Diabetes complications can be prevented through timely application of several measures such as lifestyle modification and control of blood glucose and blood pressure for diabetic patients [3,5-8].

The identification of persons with diagnosed DM in electronic medical records (EMRs) is essential to quality improvement initiatives, clinical decision support systems, and regional disease prevalence estimates used by public health departments. Although DM diagnoses have typically been captured by International Classification of Diseases (ICD) codes and stored in EMRs, previous studies found that diagnostic codes alone do not adequately represent DM diagnoses across a population, resulting in underestimates of disease prevalence and challenging the development of electronic approaches to clinical management [9,10]. The prevalence of DM in 2014 in Maine was 7.8%, whereas the codified prevalence is 6.8% in our database. It indicates a gap caused by uncodified DM in the structured EMRs of patients. Diabetic patients who have received little or no diabetes care are unlikely to be associated with a diabetes-specific diagnosis code for billing, as are patients who transfer their care between multiple unaffiliated health care systems but receive no DM care for some time. To overcome this shortcoming, manual chart reviews of unstructured clinical notes have been used to identify uncodified DM cases. However, this method involves high labor costs and long latency, which has limited use for large scale datasets [11-13].

One possible solution to the problem and a fully automated alternative and acceptable means of delivering cost-effective case finding is the use of natural language processing (NLP), a Web-based technique. NLP has increasingly been used to enhance case finding for some high-impact chronic diseases such as heart failure and cancer through analyzing narrative text in EMRs [14-16]. The advantage of the automated NLP-based case finding algorithm is that it allows for the rapid real-time identification of uncodified diagnoses from large datasets. It also allows for the rapid preprocessing of unstructured clinical notes for different diseases and clinical conditions before a diagnosis is selected [14,16]. However, the existing NLP applications are mainly based on a small sample of patients with a limited number of clinical notes. Currently, the application of NLP in public health and medicine faces the following challenges [17-21]: (1) a lack of a comprehensive knowledge base to generate the accumulated domain knowledge from the targeted patient population; (2) a lack of a comprehensive data model to encapsulate the unstructured clinical notes of various formats across different health care facilities; (3) and a lack of a robust and scalable analytics pipeline to process a large number of EMR notes across statewide health care facilities.

**Figure 1:**
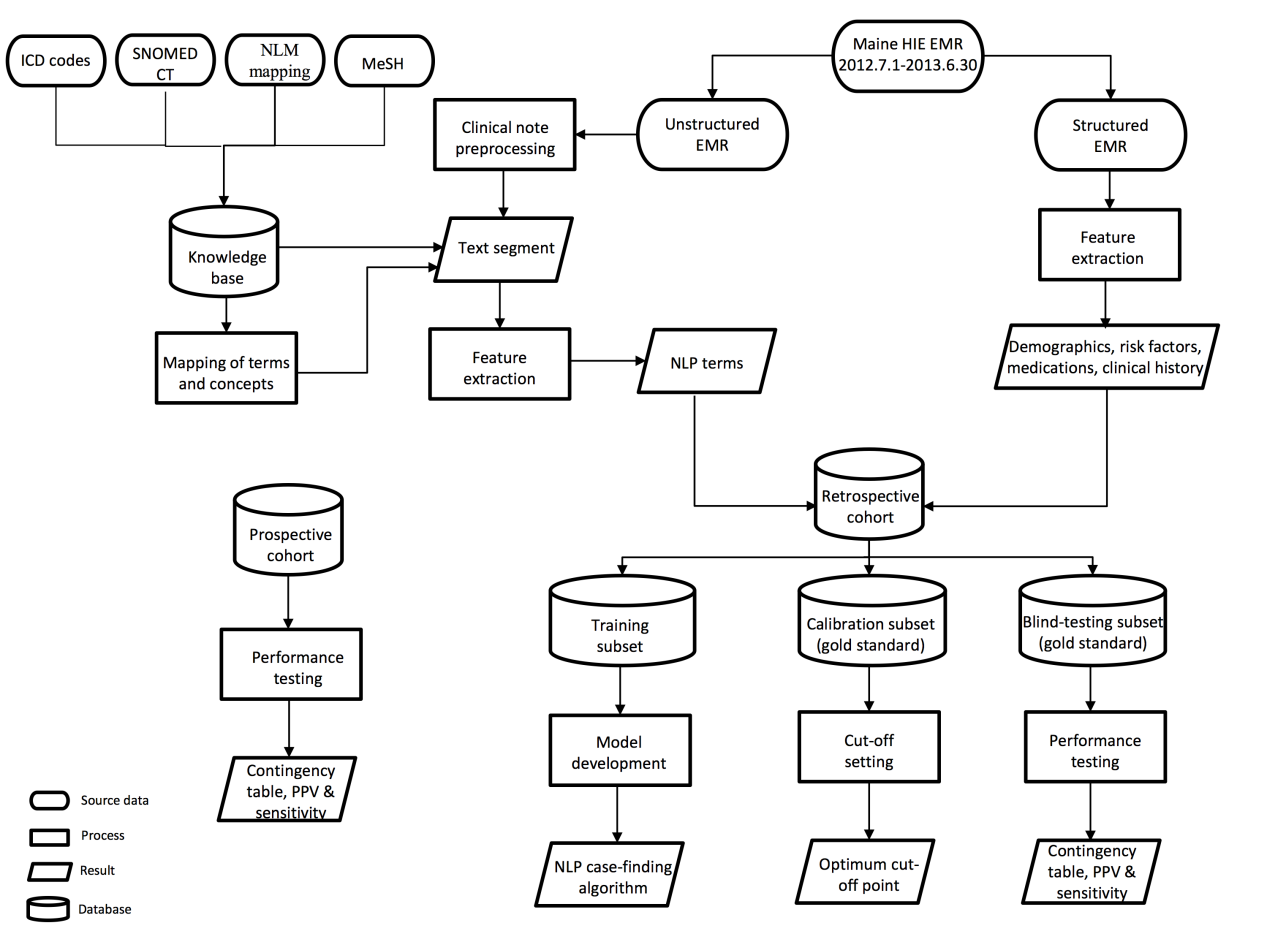
A schematic presentation of the natural language processing (NLP)–based algorithm integrated into the statewide diabetes mellitus case finding and surveillance. The clinical note was preprocessed and identified to generate the decision. The knowledge bases, statistical model, and the gold standard datasets form the basis of the NLP engine. ICD: International Classification of Diseases; NLM: US National Library of Medicine; MeSH: Medical Subject Headings; EMR: electronic medical record; HIE: health information exchange; PPV: positive predictive value. SNOMED CT: Systematized Nomenclature of Medicine – Clinical Terms.

The aim of this study was therefore to develop and integrate an online real-time NLP-based DM case finding algorithm into the health information exchange (HIE) care flow in the state of Maine, United States (Figure 1). We hypothesized that the algorithm we developed could find additional patients with DM who were not identified by codified diagnoses in structured EMRs. This algorithm was built on a knowledge base that incorporates taxonomies and controlled vocabularies encoding domain knowledge, as well as the task-oriented characteristics of clinical notes. It also used both structured and unstructured information and data available in EMRs, which were treated as variables for statistical learning in identification of uncodified DM diagnoses.

## Methods

### Ethics Statements

Protected personal health information was removed for the purpose of this research. Because this study analyzed deidentified data, it was exempted from ethics review by the Stanford University Institutional Review Board (October 16, 2014).

### Data Sources

**Figure 2:**
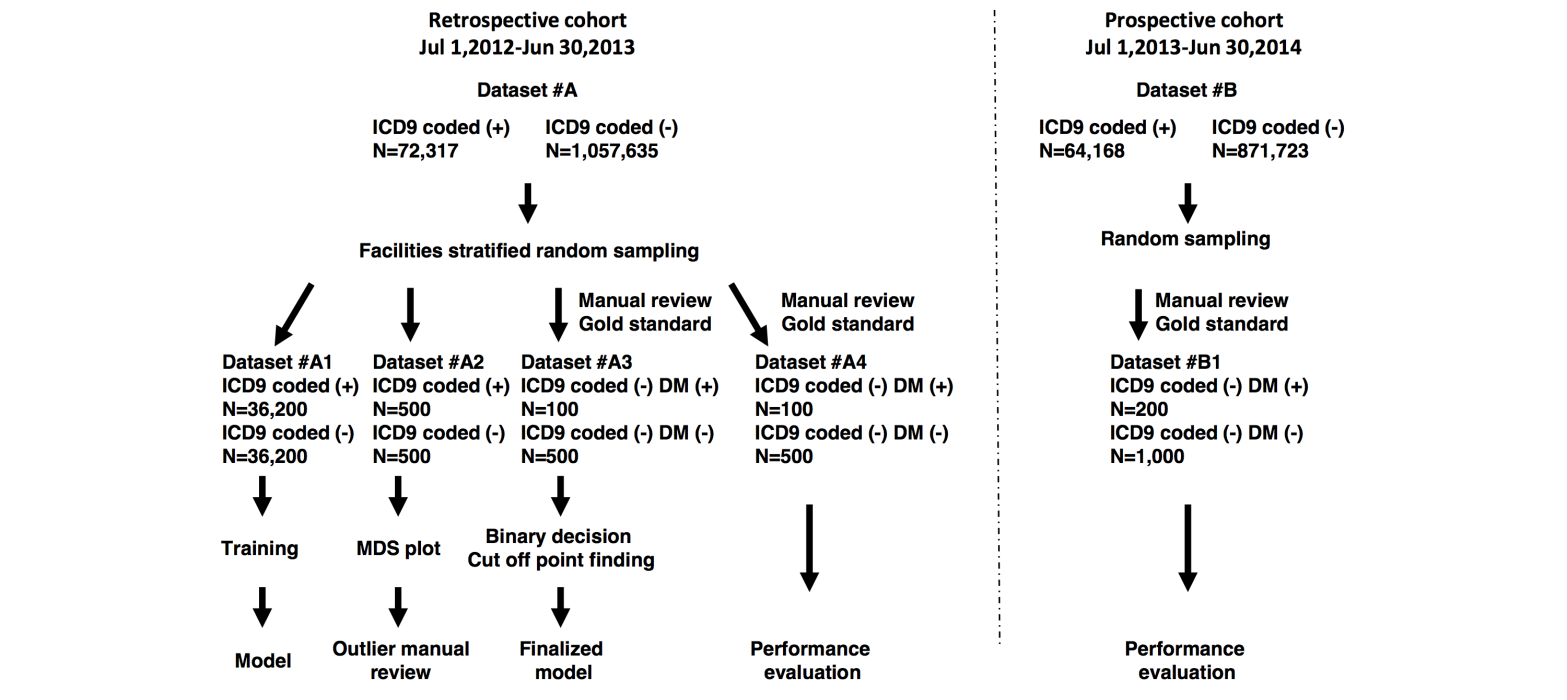
Cohort construction of the study. ICD9: International Classification of Diseases, Ninth Revision; DM: diabetes mellitus; MDS: multidimensional scaling.

Data for this study were extracted from the HIE dataset administered by HealthInfoNet—an independent nonprofit organization. The dataset contains records of nearly 95% of the population in the state of Maine. There are 35 HIE-associated hospitals, 34 federally qualified health centers, and more than 400 ambulatory practices [22,23]. To identify the DM cohort, clinical notes of all categories in the Maine HIE EMR database were analyzed. Clinical notes are also known as progress notes, which are the part of a medical record where health care professionals document the details of a patient's clinical status or achievements during the course of inpatient care or outpatient care. Clinical notes in our study are encounter based. These notes were divided into 2 subcohorts. The retrospective cohort contained 1,385,280 notes representing 1,129,952 patients covering the period from July 1, 2012, to June 30, 2013, and the prospective cohort comprised 982,211 clinical notes representing 935,891 patients recorded from July 1, 2013, to June 30, 2014 (Figure 2). Clinical notes were derived from more than 100 different types of clinical reports, including history or physical reports, discharge summaries, and emergency reports.

### Algorithm Overview

The patients with DM were defined as those who had DM noted as either primary or secondary diagnosis (International Classification of Diseases, Ninth Revision, Clinical Modification, ICD-9-CM, codes: 249, 249.x, 249.xx, 250, 250.x, and 250.xx) in their medical records [24]. The case finding algorithm consisted of 3 sequential steps based on both structured and unstructured EMR information (Figure 1). The first step involved a preprocessing of unstructured clinical notes to remove information indicating the patient did not have DM, such as family history of DM and negation (ie, the patient denied DM). This step removed the misleading information to avoid false-positive errors, thus improving the performance of subsequent steps. The second step entailed a feature extraction that mapped DM risk factors recognized in previous studies [25-29], medications extracted from Unified Medical Language System, and NLP terms into the structured metadata. In the third step, a decision tree–based model based on the retrospective cohort was developed to determine whether a patient had DM. The development procedures are detailed in later sections. To support the whole algorithm pipeline, the NLP engine was created, including knowledge base, statistical models, and gold standard datasets as functional modules. Their construction and utilization are described below.

### Knowledge Base

The knowledge base consisted of 3 cores: (1) DM-related clinical terms as the controlled vocabularies; (2) antidiabetic medications; and (3) extracted rules in the clinical notes.

Clinical terms in our NLP knowledge base were derived from the following sources: (1) the description and synonyms of ICD-9-CM codes under 249, 249.x, 249.xx, 250, 250.x, and 250.xx; (2) the comprehensive clinical terminologies within SNOMED CT (Systematized Nomenclature of Medicine – Clinical Terms) [30]; (3) a mapping of ICD-9-CM with SNOMED CT proposed by the US National Library of Medicine (NLM) [31], based on the concepts and synonyms mapped to ICD codes 249, 249.x, 249.xx, 250, 250.x, and 250.xx; (4) the headings returned by the query of “diabetes” using NLM for article indexing [32] in a controlled vocabulary thesaurus, namely, Medical Subject Headings (MeSH). These clinical terms in the knowledge base were further tokenized, combined, and filtered to derive our controlled vocabulary of single and dual tokens. If those controlled vocabularies contained stop words, for example, “the,” “a,” “of,” provided by the text mining (tm) package (R Development Core Team) [33], they were removed. In total, 742 final NLP terms were identified (Multimedia Appendix 1); of these, 72 were found to be significantly associated with DM diagnosis (Mann-Whitney test *P* value <.05) in the retrospective cohort. Here, the patients who were assigned any of the ICD-9-CM codes 249, 249.x, 249.xx, 250, 250.x, 250.xx during the encounter were defined as having a diagnosis of DM.

Antidiabetic medications were identified from the Unified Medical Language System database. Out of 36 medications analyzed, 22 were found to be significantly associated with DM diagnosis (Mann-Whitney test *P* value <.05) in the retrospective cohort.

Because information on DM risk factors (eg, body mass index or BMI, high blood pressure, obesity, smoking history, and alcohol use disorders) might be presented in multiple unstructured formats in EMRs, we developed a series of regular expressions and rules to unify unstructured information and subsequently standardize feature categories. For example, BMI could be available from clinical notes, but in many instances only height and weight were provided. The BMI was then divided into 4 categories: underweight, normal, overweight, and obesity, according to the World Health Organization classification [34]. Additionally, to make the knowledge base more compatible with the expression of clinical notes, it was updated iteratively along with development of the retrospective model.

### Preprocessing and Feature Extraction

Intuitively, DM-related words in the notes can be used to classify a DM case. However, this simpleminded note-processing method ignores negative expressions, for example, “The patient denied DM” in the note. Obviously, such negation will mislead the algorithm to wrongly classify the patient as a DM case. To avoid this kind of error, negation should be handled first before being fed into the pipeline. Preprocessing to remove family DM history is done because of similar considerations: the note with sentence “his mother had diabetes mellitus” does not classify the corresponding patient, “he,” as a diabetic patient. To ensure NLP specificity, segments associated with negation and family history of DM as described above were removed during preprocessing according to the entries in the knowledge base. The vocabulary of negation was populated using the lexicon proposed by NegEX [35]. The family-related words [36] were used to initiate the vocabulary of family history.

To break narrative text in clinical notes into smaller pieces, we applied the text semantics. A note was collapsed into paragraphs, sentences, and lines as basic units with nonoverlapping contents. Criteria to define a basic unit were developed on statistics of the text lengths and newline characters. If a paragraph (or a sentence, a line) satisfied criteria of a basic unit, it was regarded as one segment without further decomposition. The parts of speech were annotated and referred for sentence boundary detection against the confusion between periods and decimal points using openNLP (R Development Core Team) [33]. When a segment contained a word or a phrase in the vocabularies associated with negation and family history, this segment was removed from the note.

To map the unstructured text into structured metadata, the knowledge base was applied to the standardized clinical notes after preprocessing. When matching the text with the NLP terms and medications in the knowledge base was successful, the structured data of the notes were coded as “1,” otherwise as “0.” Then DM risk factors were extracted to further enrich the clinical notes metadata using the rules and regular expressions stored in the knowledge base.

### Workflow of Gold Standard Dataset

Gold standard datasets were created for model development and validation purposes (Figure 2). The datasets contained a subset of patients with or without DM. The patient DM status was determined by manual chart reviews of clinical notes conducted by 2 physician-curators. If a patient had any notes showing DM diagnosis, he or she was coded as having DM. The 2 physicians reviewed each note individually and assessed whether the note showed the presence of DM. After individual review, the 2 assessments for each note were compared. Any disagreement was discussed by the 2 physicians and an agreement was reached [37]. When there was a disagreement on diagnosis that could not be resolved by discussion between the 2 curators, the patient was excluded. The datasets created through this process were used as the gold standard to define the cutoff point, run the blind testing, or to validate our NLP-based case finding algorithm. The cohort construction of the gold standard datasets is shown in Figure 2.

### Model Development

A model was developed on the retrospective cohort (Figure 2). The clinic’s facilities where clinical notes were derived were randomly allocated to 1 of the 2 subsets: one for training and for finding the cutoff point (n=17 facilities) and the other for blind testing (n=18 facilities). Within the subsets for training and finding the cutoff point, all available notes (n=44,368) with codified DM diagnoses, and an equal number of uncodified notes (n=44,368), were selected to construct a training subcohort for model development. In the remaining uncodified subset, a gold standard dataset was constructed by randomly selecting 100 positive (DM) patients and 500 negative (non-DM) patients as the subcohort for finding the cutoff point. A further random sample of 100 positive and 500 negative patients identified from uncodified notes in the blind testing subset were selected to construct the blind testing subcohort.

**Figure 3:**
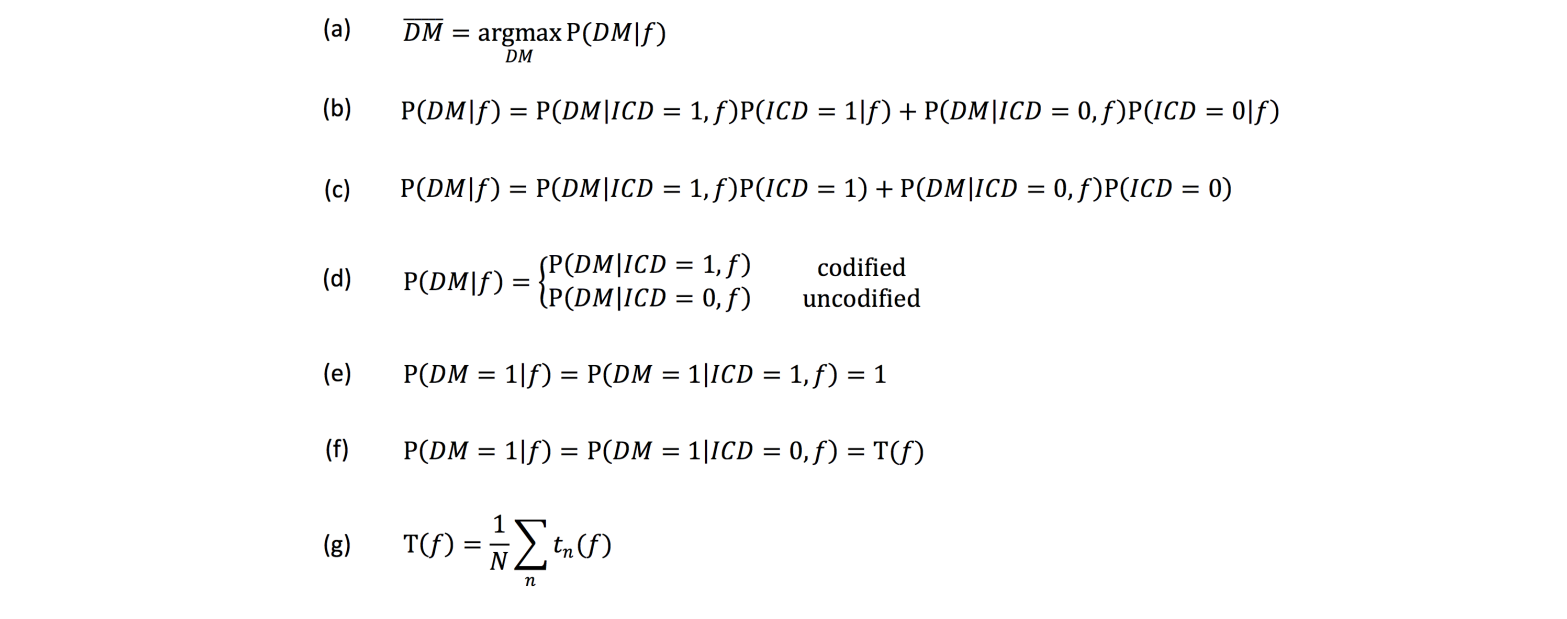
Equations describing the modeling process of the natural language processing (NLP)–based algorithm.

By feeding the training subcohort to the preprocessing and feature extraction, each note had a feature vector denoted as *f*. The identification of DM was stated as maximum a posteriori probability (MAP) estimation in Figure 3 (a), where *DM* was a binary random variable indicating whether the sample had a DM diagnosis (*DM*=1). To take diagnosis codes into consideration, a binary variable *ICD* was introduced to indicate whether a note was codified (*ICD*=1). By inserting *ICD* into the posterior and then applying the Bayesian rule, we had the decomposition in Figure 3 (b).

Because the assignment of diagnosis code was independent of the extracted feature, the model was simplified to the equation in Figure 3 (c).

The first term on the right side determined the probability of DM for a codified note, while the second term on the right side for an uncodified note. As coding information was known, we had 2 branches to obtain the posterior a shown in Figure 3 (d).

The great majority of uncodified notes did not include a DM diagnosis, while most DM codified notes were ICD-9-CM DM diagnoses. This led us to develop the following class labeling method:

1. If a note is codified, this note should have a diagnosis of DM (Figure 3 (e));

2. If a note is not codified, a model should be built to estimate the probability (Figure 3 (f)).

As a result, the inference of DM diagnosis for a codified note was only dependent on the ICD code noted in the structured data, whereas for uncodified notes we trained a random forest model [33,38] to obtain T(*f*) (Figure 3 (g)), where *t*_n_ was the *n* th decision tree in the random forest.

At the perspective of hierarchical tree, the model could be considered as a combination of a predetermined tree-based model and a random forest-based model. The predetermined tree was developed based on the ICD-9-CM diagnosis codes associated with DM, which represented human prior knowledge. The random forest-based model was developed by extracting information from clinical notes, which represented machine learning knowledge.

The model was first trained with codified notes, the DM-positive sample, and uncodified notes, the DM-negative sample. The false positives in the training sample were uncodified notes either with or without a DM diagnosis. The former was regarded as the positive sample in the next round of training. By applying the 2 steps iteratively, the model as well as the knowledge base associated with the expression of family history and negation was fine-tuned. All false-positive cases were reviewed manually to understand how these occurred.

This codified-note–driven iterative training scheme was based on the hypothesis that the notes’ features should be similar between codified notes and uncodified notes where a DM diagnosis was found. To test this hypothesis and validate the method, multidimensional scaling (MDS) plots were constructed with 1000 samples randomly selected from the training subcohort to illustrate the distribution of notes.

### Patient Classification Cutoff Point Determination

As the algorithm was developed to find out uncodified DM cases, the proportion of true positives among the identified samples, positive predictive value (PPV), was the most important indicator of performance. With a PPV of ≥90%, the proportion of false-positive cases is less than 10%. On the other hand, given that the method was to identify uncodified cases in addition to the codified cases, maintaining a high level of PPV at the expense of sensitivity is acceptable. The way we located the optimal cutoff by considering the trade-off between PPV and sensitivity was also presented in a previous NLP study [39]. Given that our algorithm assigned a classification probability to each subject, we aimed to find an optimal cutoff point to achieve the maximum classification sensitivity with a predefined PPV of 90%. To achieve a 90% PPV, the classification specificity can be calculated through a linear formula, thus forming a straight line overlaid on the receiver operating characteristic (ROC) curve. The combination of sensitivity and specificity in the region above the line allowed for a performance with >90% PPV. Thus, the cutoff point was set at the first intersection between the line and the ROC curve.

At the final stage of the retrospective model development, the case finding algorithm was blind tested on patients from health care facilities that were not included in the training subset.

### Prospective Case Finding and Validation

Our NLP-based DM case finding algorithm was then deployed online through integration into the HIE real-time population exploration dashboard system. The clinical notes (N=982,211) covering the period from July 1, 2013, to June 30, 2014, were aggregated for prospective validation of the algorithm. An independent gold standard dataset was constructed based on chart reviews of clinical notes of 200 patients with DM and 1000 patients without DM randomly selected from the prospective cohort (Figure 2). The prospective classification performance on the gold standard dataset was evaluated using the following parameters: PPV, sensitivity, specificity, negative predictive value (NPV), and the area under the ROC curve. A total of 200 samples were further randomly selected from the uncodified DM cases identified by the algorithm to evaluate the case finding accuracy on the entire prospective cohort. On the basis of the longitudinal records of both clinical notes and diagnosis codes for each patient in the HIE EMR database, a temporal comparison of the 2 sources was analyzed.

## Results

### Case Finding Algorithm Performance

**Figure 4:**
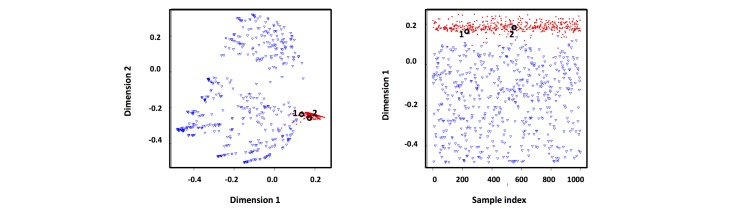
The multidimensional scaling (MDS) plots of the training result. This analysis was aimed at detecting meaningful underlying dimensions, for example, 1 and 2, which allow the explanation of the observed similarities (distances) between the investigated subjects. The axes of the MDS plots represent no real sizes and thus were marked as dimension 1 and dimension 2 without units. The red dots and blue triangles, indicating the positive and negative samples, were clearly separated. The “false positives” are circled in the plot. Chart reviews showed that these were notes with a genuine diagnosis of diabetes mellitus.

An MDS plot was constructed to visualize the classification performance. As shown in Figure 4, out of 500 uncodified notes, 2 were classified as DM diagnosis. A closer examination revealed that these “false-positive” cases had notes with genuine diagnosis of DM. This MDS plot indicated that (1) our model effectively differentiated the notes from those patients with DM diagnosis and those without DM diagnosis and (2) our NLP-based analysis of clinical notes can identify uncodified notes with diagnosis of DM.

Figure 4 shows that more than 99% of the uncodified notes were linked to patients without DM diagnosis and more than 99% of the codified notes were linked to patients with DM diagnosis. There were only 1% mislabeled samples in the training dataset, which did not alter the model performance [40].

### Diabetes Mellitus Discriminant Variables

**Figure 5:**
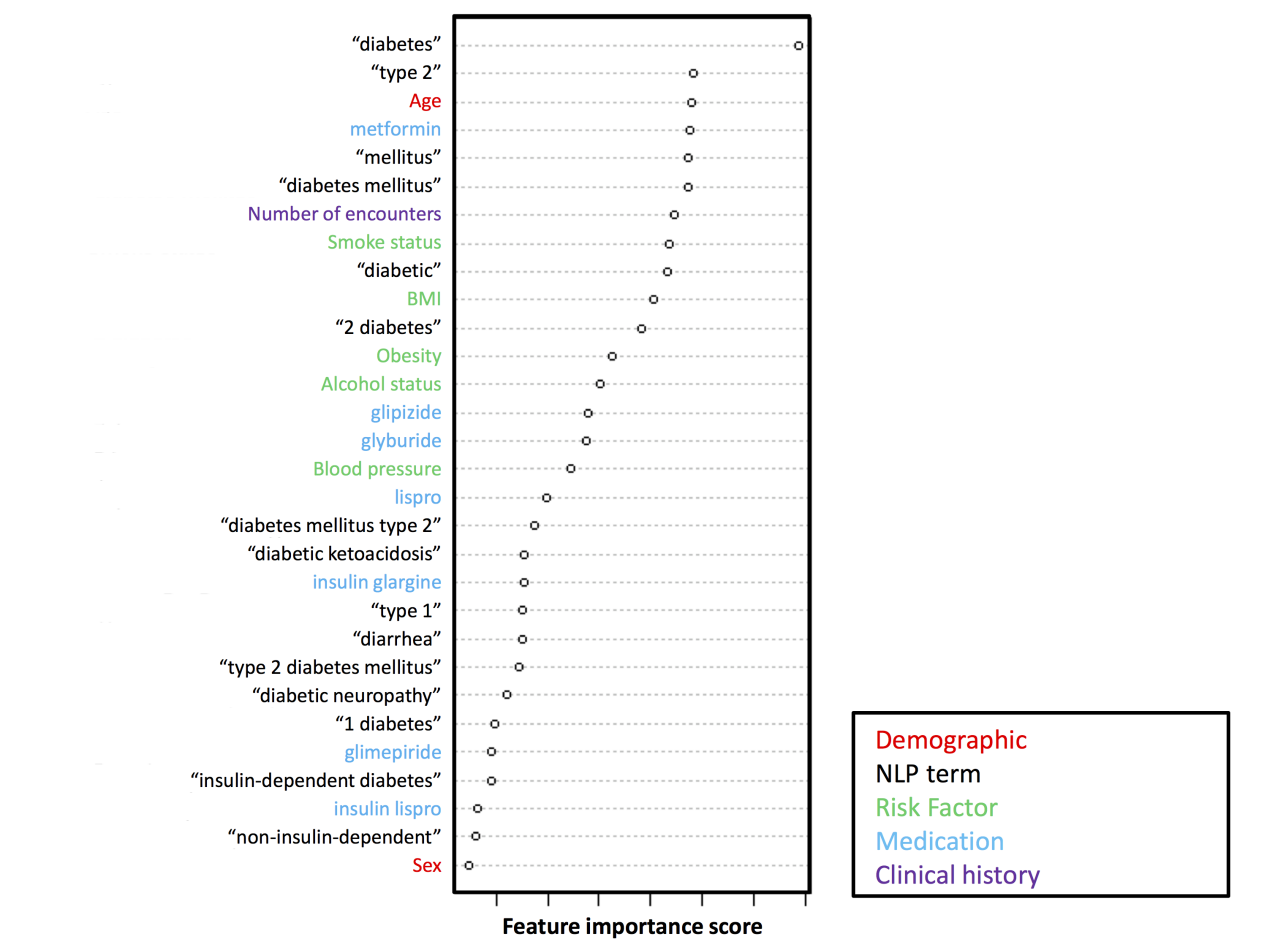
List of the top 30 clinical variables included in the diabetes mellitus natural language processing (NLP)–based model. BMI: body mass index.

A total of 100 DM discriminant features were retained in the final model, including demographics (n=2), risk factors (n=5), clinical history (n=1), medications (n=20), and NLP-extracted clinical terms (n=72; Multimedia Appendix 1). Figure 5 shows the top 30 features ranked by their importance in the model. The importance of each feature was rated according to the mean decrease in algorithm accuracy scaled by standard deviation after randomly permuting the variable values. A higher mean decrease in accuracy (node impurities from splitting on the variables; specifically, the node impurity is measured by the Gini index) corresponds to greater importance of the feature [40]. Among the top 30 features, “diabetes” and “type 2,” which directly indicate DM, were the top 2 features, followed by age, an important predictor of DM [41,42], and then “metformin,” a first-line antidiabetic drug. The remaining important discriminant features were high blood pressure, cigarette smoking, history of alcohol use, BMI, and “obesity.”

### Patient Classification Cutoff Point Determination

The decision tree–based classification scores were evaluated to determine a cutoff point that allows maximal sensitivity with a ≥90% PPV (Multimedia Appendix 2). With this cutoff value (set as .618), the continuous classification scoring outputs were converted to reach a binary decision to identify genuine DM cases.

### Retrospective Blind Testing

**Figure 6:**
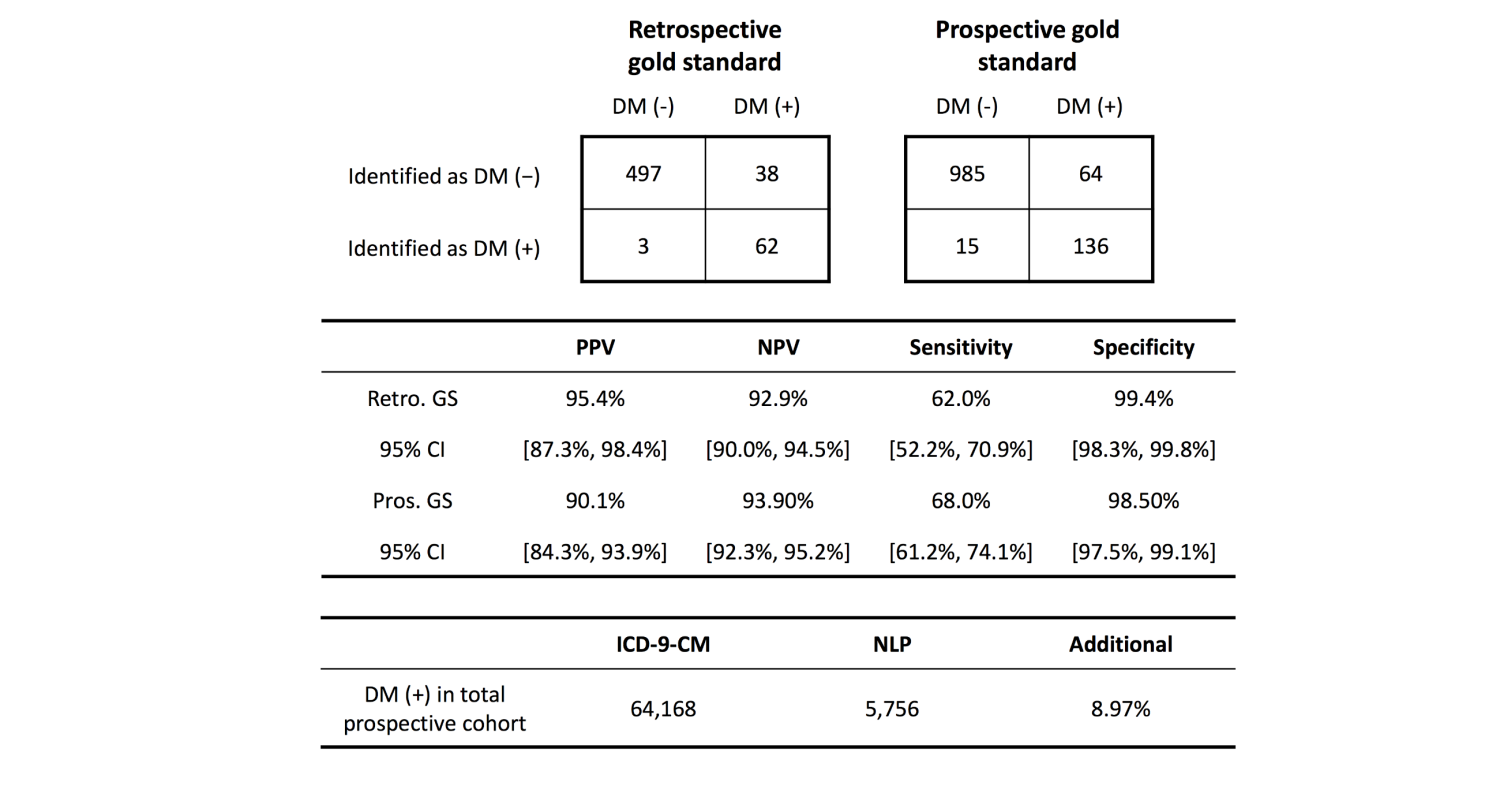
Performance evaluation of the proposed case finding algorithm. Top: the contingency tables on blind test and prospective gold standard datasets. Middle: the positive predictive value (PPV), negative predictive value (NPV), sensitivity, and specificity of the validation based on the retrospective blind testing subcohort and prospective cohort. Bottom: the prospective case finding results in the total population. DM: diabetes mellitus; GS: gold standard; ICD-9-CM: International Classification of Diseases, Ninth Revision, Clinical Modification; NLP: natural language processing.

As shown in Figure 6, in the retrospective blind testing, our NLP-based analysis achieved a 95.4% (62/65) PPV, 62.0% (62/100) sensitivity, 99.4% (497/500) specificity, and 92.9% NPV (497/535). The blind testing results indicate that the knowledge acquired from some hospital facilities could be leveraged to allow prediction in others (eg, learning transfer) [43].

### Prospective Validation

The prospective performance of the algorithm was explored by chart review over a gold standard dataset consisting of randomly selected 200 patients with DM and 1000 patients without DM in the uncodified subcohort (Figure 2). The PPV was 90.1% (136/151), which was within the 95% CI of the retrospective blind testing PPV (87.3%-98.4%). The sensitivity was 68.0% (136/200). The specificity, NPV, and area under ROC curve were 98.50% (985/1000), 93.90% (985/1049), and .929, respectively (Figure 6).

The algorithm was deployed to allow real-time DM case finding on the entire prospective cohort. A total of 64,168 patients with DM were identified from codified DM diagnosis, while our NLP-based algorithm identified an additional 5756 patients, resulting in an 8.97% (5756/64,168) increase in the total patients with DM during the study period. To further explore the case finding accuracy, we randomly selected 200 samples from the 5756 samples. Manual review showed that of the 200 samples there were 183 DM cases and 17 normal patients, resulting in an accuracy of 91.5% (183/200). Such accuracy was above the predetermined PPV (90%) in the calibration phase and was within the 95% CI of the retrospective blind testing PPV (87.3%-98.4%). The consistency of performance shows that it is reasonable to use the results obtained on smaller samples to reflect the performance of the algorithm on a large population.

### Temporal Comparison

The time point when a patient’s first DM diagnosis was identified by ICD codes was evaluated and compared with the time point when the DM was identified by NLP case finding algorithm. Out of 21,720 patients with DM identified by both methods, 6616 patients (6616/21,720, 30.46%) were identified by the NLP-based algorithm before a DM ICD code was noted in the medical record (mean time difference = 48 days). In particular, 19.86% (1314/6616) of patients were identified by NLP case finding 3 months or more before they were identified by a DM ICD code (Multimedia Appendix 3).

## Discussion

### Principal Findings

To the best of our knowledge, this is the first online deployment of a real-time NLP-based case finding method for DM, using both patients’ structured (eg, codified diagnosis) and unstructured (free text) clinical histories from a statewide EMR database. Consistent with our hypothesis, during a 1-year period (from July 1, 2013, to June 30, 2014), our algorithm identified 5756 additional patients with DM (an 8.97% increase in the total patients with DM) who were otherwise left undiagnosed when only code-based case finding was applied. Our finding indicates that the proportion of false negatives decreased using the NLP-based approach compared with the existing ICD-based approach (*P*<.01). Many patients with DM who were misclassified as patients without DM by the code-based case finding were correctly identified by our NLP text searching algorithm, resulting in a more complete ascertainment of DM diagnoses.

There exist several reasons why patients with diagnosed DM may have not been associated with a DM diagnosis code. Among the uncodified DM patients we identified, 30% had DM noted as secondary, discharged, or other types of diagnosis and 63% had a history of diabetes in clinical records. A possible reason for missing diagnostic codes in those cases might be that if a patient was admitted to the hospital owing to more acute or life-threatening clinical conditions, information related to DM was overlooked when ICD coding was conducted. Therefore, there is a strong need for enhancing the current ICD coding practice in hospitals and other health care facilities in the state of Maine to ensure that all DM diagnoses noted in the patients’ medical records are coded.

### Strengths and Limitations

Although several standardized coding systems (eg, ICD, Logical Observation Identifiers Names and Codes) have been used to record diagnoses, procedures, laboratory tests, and medications associated with each patient encounter, a large amount of information related to patients’ clinical histories were also available in the form of unstructured free text in EMRs. In addition to the terms directly describing DM (eg, “diabetic,” “type 1,” “diabetes mellitus”), our NLP algorithm was able to obtain more complete medical histories based on information about risk factors and medications available from clinical notes. A range of conventional DM risk markers (eg, age, smoking, BMI, and blood pressure) [42,44-46], emerging risk markers (eg, overweight) [47], and antidiabetic drugs (eg, metformin) were identified and used to enhance DM case detection. In particular, metformin, the first-line medication for type 2 diabetes, appeared to be the most important drug in our feature selection process. These findings indicate that our algorithm effectively incorporated a variety of clinically relevant features, leading to a significant improvement in DM case finding in the population of the state of Maine.

Another strength of our NLP case finding algorithm is the ability to find uncodified DM cases before the assignment of ICD-9-CM codes. The proposed DM case finding methodology used NLP algorithm in parallel with ICD-9-CM codes. In the prospective study, 69,924 patients with DM were identified. Among those 69,924 patients, 21,720 patients were able to be identified by both methods. That is, there were 21,720 DM patients having clinical notes that indicated they had DM. 30.46% (6616/21,720) of those patients had such clinical notes associated with an encounter earlier than the assignment of a DM diagnosis code, while 69.54% (15,104/21,720) of those patients had such clinical notes during the same encounter when a DM diagnosis code was given. Compared with using ICD-9-CM codes alone, the NLP algorithm was able to identify 30.46% (6616/21,720) of patients with DM at an earlier encounter, giving a mean time difference of 48 days. More importantly, a significant proportion of these patients (1314/6616, 19.86%) were identified 3 months or more before a DM diagnosis code was noted. For those patients, this time period is sufficient to initiate aggressive lifestyle interventions that have a long-term impact to delay progression and prevent complications of diabetes [48]. Thus, this early detection capability is clearly an advantage of our DM NLP algorithm such that these high-risk individuals can be selected for timely initiation of targeted prevention, care, and treatment.

We noted that there are some limitations in our study. First, although the use of statistical learning improved the performance of the case finding algorithm, it has inevitable misclassification errors. There were a couple of DM cases located close to the “borderline,” that is, the cutoff point for the algorithm to differentiate between DM cases and normal samples. The DM cases with outputs closed to the cutoff point for the algorithm were those who were susceptible to misclassification errors, compromising false negatives. DM cases at borderline represented DM patients with incomplete DM feature profile, that is, patients having no DM-related risk factors or medication records but having clinical notes confirming DM diagnosis, or patients having no DM-related risk factors or clinical notes but having medication records. Such incomplete profiles could mislead the algorithm. Second, the relatively small sample size of the “gold standard” dataset introduced the possibility that some relatively rare clinical phenotypes of DM—where clinicians documented diabetes in a nonstandard way—might not be accounted for during model training. Third, we were unable to identify whether the patients with DM found by the NLP algorithm were those with newly diagnosed DM or those with a long-standing diagnosis. Fourth, we acknowledge our case finding method’s limitation that it depends on the physician’s diagnosis of the disease and the documentation quality in clinical notes. Finally, the model was developed on the patient data in the state of Maine. Extra risk factors such as sociodemographic factors may need to be considered for adjustment purpose when this learning is transferred and applied to other geographic regions.

### A Web-based Identification Tool

Our NLP algorithm has been deployed online through integration into the Maine State HIE workflow, currently allowing real-time statewide identification of patients with uncodified DM. It provides doctors, hospitals, and other providers in the state HealthInfoNet network with an effective online utility to achieve a more complete assessment of the DM burden in their location. Incorporating the DM case finding algorithm with the existing health care system makes the best use of information available in EMRs. Together with the previously successful integration of our other NLP case finding algorithms, including that for congestive heart failure [14], there is a strong potential to expand the application of this novel method to enhance case finding for other diseases in Maine and other states in the United States and in other countries.

### Conclusions

Our NLP-based DM case finding algorithm was developed and validated on a population-based dataset in the state of Maine. The results strongly support our hypothesis that the NLP-based algorithm could identify additional patients with DM to complement the existing ICD-code–based case finding method. Online real-time integration of our DM case finding algorithm into the Maine HIE workflow can enhance DM case detection and facilitate efforts toward timely initiation of targeted management and care for patients with DM. From the patient’s perspective, many patients with DM across the state of Maine, who were not identified from ICD codified diagnosis, would benefit from information we provide by being able to take initiatives to seek care and plan their personal strategies to monitor and control their diabetes status. In this regard, our online real-time DM case finding utility not only benefits all stakeholders including payers, providers, and policy makers in the Maine health care system, but also serves as a demonstrative Web-based project for future application to improve DM case finding for targeted care and treatment in other states and countries, making a contribution to alleviate the DM burden.

## Appendix

Multimedia Appendix 1 A list of 100 discriminant features used by the final model as well as the feature importance, and a list of 742 natural language processing terms used by the initial modeling process.

Multimedia Appendix 2 Determination of the cutoff point of the subject classification probabilities. Top: the receiver operating characteristic curve and the line determined by the prevalence and 90% positive predictive value were intersected at the cutoff point. Bottom: their intersection (dashed rectangle) is magnified and indicated by the circle.

Multimedia Appendix 3 The distribution of patients by the time intervals between natural language processing–based diabetes mellitus identification and codified diagnosis.

## Abbreviations

BMI: body mass index
DM: diabetes mellitus
EMR: electronic medical record
HIE: health information exchange
ICD: International Classification of Diseases
ICD-9-CM: International Classification of Diseases, Ninth Revision, Clinical Modification
MDS: multidimensional scaling
MeSH: Medical Subject Headings
NLM: US National Library of Medicine
NLP: natural language processing
NPV: negative predictive value
PPV: positive predictive value
ROC: receiver operating characteristic
SNOMED CT: Systematized Nomenclature of Medicine – Clinical Terms
