# Supplementary material for: Web-based Real-Time Case Finding for the Population Health Management of Patients With Diabetes Mellitus: A Prospective Validation of the Natural Language Processing–Based Algorithm With Statewide Electronic Medical Records
Source: JMIR Med Inform. 2016 Nov 11;4(4):e37. doi: 10.2196/medinform.6328 (PMC5124114; doi:10.2196/medinform.6328)
Supplement: Multimedia Appendix 1 [file medinform_v4i4e37_app1.pdf]

Multimedia Appendix 1. A list of 100 discriminant features used by the final model as well as the feature importance, and a list of 742 NLP terms used by the initial modelling process.

Demographics (N=2)

Age
Gender

Risk factors (N=5)

Smoking history
Alcohol use disorders
Obesity
BMI
High blood pressure

Clinical history (N=1)

Encounter counts

Medications (N=20)

pramlintide
glipizide
lispro
nph insulin
exenatide
insulin glargine
insulin lispro
nateglinide
repaglinide
sitagliptin
metformin
acarbose
aspart protamine
insulin aspart
eplerenone
glimepiride
pioglitazone
rosiglitazone
glyburide
glulisine

NLP terms (N=72)

diabetes
type 2
mellitus
diabetes mellitus
diabetic
2 diabetes
diabetes mellitus type 2
diabetic ketoacidosis
type 1
diarrhea
type 2 diabetes mellitus
diabetic nephropathy
1 diabetes
insulin-dependent diabetes mellitus
non-insulin-dependent
gangrene
250.50

diabetic retinopathy
diabetic foot
250.00
type ii
ii diabetes
non-insulin dependent
1 diabetic
cystic fibrosis
glycosuria
iddm
diabetic foot ulcer
type ii diabetes mellitus
diabetic peripheral
diabetic neuropathy
diabetic peripheral neuropathy
non-insulin dependent diabetes mellitus
mixed hyperlipidemia
type i
retinal detachment
diagnosed diabetes
newly diagnosed diabetes
chronic diabetic
irma
ketonuria
i diabetes
diabetes mellitus type 1
niddm
brittle diabetes
hypoglycemic event
type 1 diabetes mellitus
diabetes mellitus with neuropathy
diabetes mellitus type ii
acanthosis nigricans
brittle type
insulin treated
insulin dependent diabetes mellitus
diabetic ophthalmoplegia
type i diabetes mellitus
nonproliferative diabetic retinopathy
proliferative diabetic retinopathy
labile diabetes
diabetic polyneuropathy
diabeticorum
photocoagulation
diabetic renal
mellitus poorly
diet controlled diabetes mellitus
ischemic ulcer
non-insulin-dependent diabetes mellitus
painful diabetic
necrobiosis lipoidica diabeticorum
mellitus well
ischemic diabetic
diabetic autonomic
diabetic skin

Feature importance of the 100 variables

X**Variables**	**Importance in the model**
diabetes	18980.1
type 2	2539.263
Age	2410.172
metformin	2321.873
mellitus	2271.471
diabetes mellitus	2243.418
Encounter counts	1726.965
Smoking history	1587.573
diabetic	1519.961
BMI	1174.337
2 diabetes	903.3761
Obesity	522.418
Alcohol use disorders	414.8557
glipizide	322.9788
glyburide	317.946
High blood pressure	230.0586
lispro	143.9476
diabetes mellitus type 2	116.9359
diabetic ketoacidosis	94.08296
insulin glargine	92.93966
type 1	92.5705
diarrhea	90.47746
type 2 diabetes mellitus	84.24551
diabetic nephropathy	67.82308
1 diabetes	52.82358
glimepiride	50.82773
insulin-dependent diabetes mellitus	49.45513
insulin lispro	37.92022
non-insulin-dependent	36.97055
Sex	31.95784
gangrene	28.86763
250.5	27.68812
diabetic retinopathy	19.9091
diabetic foot	17.95995
250	15.52397
nph insulin	12.95181
acarbose	10.43844
insulin aspart	9.423584
type ii	9.42223

pioglitazone	9.133104
ii diabetes	7.922319
non-insulin dependent	6.315834
1 diabetic	6.001132
cystic fibrosis	5.266337
sitagliptin	4.06094
glycosuria	4.028382
iddm	3.622388
diabetic foot ulcer	3.61608
type ii diabetes mellitus	2.921693
diabetic peripheral	2.673623
diabetic neuropathy	2.666376
diabetic peripheral neuropathy	2.271212
non-insulin dependent diabetes mellitus	2.157529
mixed hyperlipidemia	1.964269
type i	1.716569
retinal detachment	1.653132
diagnosed diabetes	1.39973
newly diagnosed diabetes	1.325211
chronic diabetic	1.31803
irma	1.095029
ketonuria	1.005114
i diabetes	0.932771
diabetes mellitus type 1	0.924317
niddm	0.911711
rosiglitazone	0.763531
exenatide	0.697849
brittle diabetes	0.682451
hypoglycemic event	0.663893
type 1 diabetes mellitus	0.647721
nateglinide	0.631116
diabetes mellitus with neuropathy	0.572632
glulisine	0.47036
aspart protamine	0.425789
diabetes mellitus type ii	0.336795
acanthosis nigricans	0.315955
brittle type	0.214094
insulin treated	0.152167
insulin dependent diabetes mellitus	0.131955
eplerenone	0.09404
diabetic ophthalmoplegia	0.080033

type i diabetes mellitus	0.077895
nonproliferative diabetic retinopathy	0.064636
proliferative diabetic retinopathy	0.063345
repaglinide	0.029219
labile diabetes	0.022087
diabetic polyneuropathy	0.018121
pramlintide	0.014572
diabeticorum	0.009039
photocoagulation	0.008781
diabetic renal	0.00712
mellitus poorly	0.006118
diet controlled diabetes mellitus	0.005275
ischemic ulcer	0.004199
non-insulin-dependent diabetes mellitus	0.003516
painful diabetic	0.00336
necrobiosis lipoidica diabeticorum	0.001604
mellitus well	0.001165
ischemic diabetic	0.00047
diabetic autonomic	0.000447
diabetic skin	0.0000106

NLP terms (N=742) used by the initial modelling process

249.00
diabetes
mellitus
secondary endocrine
endocrine diabetes
diabetes mellitus
249.10
malnutrition-related diabetes
249.40
249.70
249.80
protein-deficient diabetes
secondary diabetes
pancreatic diabetes
249.90
posttransplant diabetes
250.00
abnormal metabolic
brittle diabetes
brittle type
type 2
2 diabetes
type ii
ii diabetes
autosomal dominant
genetic defect
insulin action
pregnancy childbirth
non-steroid drugs
diabetes-pancreatic
exocrine dysfunction
diabetic
dm
dm diabetes
foot abnormality
abnormality diabetes
diabetes related
glucose
glucose tolerance
herrmann syndrome
insulin treated
non-insulin dependent
non-insulin-dependent
labile diabetes
autoimmune diabetes
insulinopenic diabetes
youth type
young type
type 1
type 10
type 11
type 3
type 4
type 5
type 6
type 7
type 8
type 9
mendenhall syndrome
diabetes glucokinase-related
mody1
mody10
mody11
mody2
mody3
mody4
mody5
mody6

mody7
mody8
mody9
ncdmm
diagnosed diabetes
niddm
mellitus syndrome
pre-existing diabetes
rabson-mendenhall syndrome
rogers syndrome
mellitus well
unstable diabetes
type i
iddm
1 diabetes
i diabetes
250.02
mellitus poorly
250.03
250.10
diabetic acidosis
diabetic ketoacidosis
250.11
250.20
honks
honks diabetic
coma associated
250.21
250.23
250.30
diabetic coma
hypoglycaemic coma
insulin coma
insulin shock
hypoglycemic coma
ketoacidotic coma
250.31
250.40
diabetic nephropathy
diabetic renal
250.41
250.50
advanced diabetic
diabetic maculopathy
diabetic retinal
background diabetic
diabetic retinopathy
bdr background
diabetic cataract
diabetic disc
diabetic eye
diabetic intraretinal
diabetic iritis
diabetic oculopathy
diabetic ophthalmoplegia
diabetic optic
diabetic papillopathy
retinal microaneurysm
diabetic traction
traction retinal
retinal detachment
vitreous haemorrhage
diffuse diabetic
early pdr
exudative maculopathy
focal diabetic
photocoagulation
inactive pdr
irma
ischaemic diabetic

ischemic diabetic
npdr
eye diabetic
preproliferative diabetic
eye proliferative
threatening diabetic
pappillopathy
pdr iris
pdr new
pdr nvd
pdr nve
preproliferative retinopathy
retinal abnormality
severe npdr
250.51
250.60
painful diabetic
diabetic neuropathy
asymmetric diabetic
diabetic proximal
diabetic amyotrophy
diabetic asymmetric
diabetic autonomic
diabetic chronic
diabetic distal
diabetic femoral
diabetic mixed
diabetic mononeuritis
diabetic mononeuropathy
diabetic motor
diabetic neurologic
diabetic neuropathic
diabetic peripheral
diabetic polyneuropathy
diabetic pseudotabes
diabetic radiculopathy
diabetic sensory
diabetic truncal
myasthenic syndrome
symmetric diabetic
1 diabetic
250.61
250.70
circulatory disorder
250.71
gangrene
250.80
appenheim's disease
ballinger-wallace
diabeticorum
bullosis diabeticorum
diabetic foot
cystic fibrosis
genetic syndrome
hormonal aetiology
hormonal etiology
diabetes deafness
diabetic bulla
diabetic cheirarthropathy
diabetic cheiroarthropathy
diabetic cheiropathy
diabetic dermopathy
diabetic dyslipidemia
diabetic hand
diabetic necrobiosis
diabetic rubeosis
diabetic scleredema
diabetic skin
diabetic thick
skin syndrome

diabetic xanthoma
diarrhea
diarrhoea
didmoad diabetes
didmoad syndrome
receptor ab
erectile dysfunction
fibrocalculous pancreatic
genetic syndromes
hyperglycaemic crisis
hyperglycemic crisis
hyperproinsulinaemia
hyperproinsulinemia
hypoglycaemic event
hypoglycaemic state
hypoglycemic event
hypoglycemic state
acanthosis nigricans
ischaemic ulcer
ischemic ulcer
lipodystrophic diabetes
hypoglycaemic warning
hypoglycemic warning
mixed hyperlipidaemia
mixed hyperlipidemia
nld necrobiosis
chronic diabetic
oppenheim-urbach disease
soft tissue
wolfram syndrome
xanthoma diabeticorum
250.81
diabetic erectile
diarrhea syndrome
diarrhoea syndrome
250.90
diabetes melliltus
250.91
secondary endocrine diabetes mellitus
malnutrition-related diabetes mellitus with ketoacidosis
malnutrition-related diabetes mellitus with renal complications
malnutrition-related diabetes mellitus with peripheral circulatory complications
diabetes mellitus associated with pancreatic disease
malnutrition-related diabetes mellitus protein-deficient
malnutrition-related diabetes mellitus without complications
protein-deficient diabetes mellitus
secondary diabetes mellitus
secondary pancreatic diabetes mellitus
malnutrition-related diabetes mellitus with multiple complications
posttransplant diabetes mellitus
abnormal metabolic state in diabetes mellitus
acrorenal field defect ectodermal dysplasia and lipoatrophic diabetes
brittle diabetes mellitus
brittle type 2 diabetes mellitus
brittle type ii diabetes mellitus
diabetes mellitus adult onset
diabetes mellitus autosomal dominant type ii
diabetes mellitus due to genetic defect in beta cell function
diabetes mellitus due to genetic defect in insulin action
diabetes mellitus in mother complicating pregnancy childbirth and/or puerperium
diabetes mellitus in mother complicating pregnancy childbirth or puerperium
diabetes mellitus induced by non-steroid drugs
diabetes mellitus induced by non-steroid drugs without complication
diabetes mellitus type 2
diabetes mellitus type 2 in nonobese
diabetes mellitus type 2 in obese
diabetes mellitus type ii
diabetes mellitus without complication
diabetes-pancreatic exocrine dysfunction syndrome
diabetic on diet only

diabetic on oral treatment
diet controlled diabetes mellitus
dm diabetes mellitus
dm induced by non-steroid drug
foot abnormality diabetes-related
glucose tol. test diabetic
glucose tolerance test indicates diabetes mellitus
insulin treated non-insulin dependent diabetes mellitus
insulin treated type 2 diabetes mellitus
insulin treated type ii diabetes mellitus
insulin-treated non-insulin-dependent diabetes mellitus
labile type ii diabetes mellitus
latent autoimmune diabetes mellitus in adult
lipodystrophy partial with reiger anomaly short stature and insulinopenic diabetes mellitus
lipodystrophy partial with rieger anomaly short stature and insulinopenic diabetes mellitus
maturity onset diabetes in youth type 2
maturity onset diabetes in youth type ii
maturity onset diabetes mellitus
maturity onset diabetes of the young type 1
maturity onset diabetes of the young type 2
maturity-onset diabetes of the young
maturity-onset diabetes of the young type 10
maturity-onset diabetes of the young type 11
maturity-onset diabetes of the young type 3
maturity-onset diabetes of the young type 4
maturity-onset diabetes of the young type 5
maturity-onset diabetes of the young type 6
maturity-onset diabetes of the young type 7
maturity-onset diabetes of the young type 8
maturity-onset diabetes of the young type 9
megaloblastic anaemia thiamine-responsive with diabetes mellitus and sensorineural deafness
megaloblastic anemia thiamine-responsive with diabetes mellitus and sensorineural deafness
mody maturity onset diabetes glucokinase-related
mody maturity onset diabetes in youth type 2
mody maturity onset diabetes in youth type ii
muscular atrophy ataxia retinitis pigmentosa and diabetes mellitus
newly diagnosed diabetes
niddm in obese
niddm insulin-treated non-insulin-dependent diabetes mellitus
niddm non-insulin dependent diabetes mellitus
non-insulin dependent diabetes mellitus
non-insulin dependent diabetes mellitus with arthropathy
non-insulin-dependent diabetes mellitus
non-insulin-dependent diabetes mellitus without complication
on subcutaneous insulin for diabetes mellitus
photomyoclonus diabetes mellitus deafness nephropathy and cerebral dysfunction
pineal hyperplasia and diabetes mellitus syndrome
pineal hyperplasia insulin-resistant diabetes mellitus and somatic abnormalities
pre-existing diabetes mellitus in pregnancy
pre-existing diabetes mellitus non-insulin-dependent
pre-existing type 2 diabetes mellitus
pre-existing type 2 diabetes mellitus in pregnancy
pregnancy and niddm
pregnancy and non-insulin-dependent diabetes mellitus
pregnancy and type 2 diabetes mellitus
type 2 diabetes mellitus
type 2 diabetes mellitus in nonobese
type 2 diabetes mellitus in obese
type 2 diabetes mellitus well controlled
type 2 diabetes mellitus with arthropathy
type 2 diabetes mellitus without complication
type ii diabetes mellitus
type ii diabetes mellitus well controlled
type ii diabetes mellitus with arthropathy
type ii diabetes mellitus without complication
unstable diabetes mellitus
unstable type ii diabetes mellitus
diabetes mellitus type 1
diabetes mellitus type i
iddm insulin-dependent diabetes mellitus

insulin dependent diabetes mellitus
insulin-dependent diabetes mellitus
juvenile onset diabetes mellitus
type 1 diabetes mellitus
type i diabetes mellitus
type ii diabetes mellitus poorly controlled
type ii diabetes mellitus uncontrolled
type 1 diabetes mellitus uncontrolled
type i diabetes mellitus poorly controlled
type i diabetes mellitus uncontrolled
diabetes mellitus with ketoacidosis
ketoacidosis in diabetes mellitus
ketoacidosis in insulin-dependent diabetes mellitus
ketoacidosis in juvenile-onset type diabetes mellitus
ketoacidosis in type 1 diabetes mellitus
ketoacidosis in type i diabetes mellitus
diabetes mellitus adult onset with hyperosmolar coma
diabetes mellitus with hyperosmolar coma
diabetic hyperosmolar non-ketotic state
honks diabetic hyperosmolar non-ketotic state
hyperosmolar coma associated with diabetes mellitus
hyperosmolar non-ketotic state in type 2 diabetes mellitus
type 2 diabetes mellitus with hyperosmolar coma
diabetes mellitus juvenile type with hyperosmolar coma
type 1 diabetes mellitus with hyperosmolar coma
hyperosmolality due to uncontrolled type 1 diabetes mellitus
coma associated with diabetes mellitus
coma associated with malnutrition-related diabetes mellitus
diabetic acidosis with coma
diabetic coma with ketoacidosis
diabetic severe hyperglycaemia
diabetic severe hyperglycemia
hypoglycaemic coma in diabetes mellitus
hypoglycaemic coma in type 1 diabetes mellitus
hypoglycaemic coma in type i diabetes mellitus
hypoglycemic coma in diabetes mellitus
hypoglycemic coma in type 1 diabetes mellitus
hypoglycemic coma in type i diabetes mellitus
ketoacidotic coma in type 2 diabetes mellitus
ketoacidotic coma in type ii diabetes mellitus
non-insulin dependent diabetes mellitus with hypoglycaemic coma
non-insulin dependent diabetes mellitus with hypoglycemic coma
non-ketotic non-hyperosmolar coma associated with diabetes mellitus
type 2 diabetes mellitus with hypoglycaemic coma
type 2 diabetes mellitus with hypoglycemic coma
type ii diabetes mellitus with hypoglycaemic coma
type ii diabetes mellitus with hypoglycemic coma
hypoglycaemic coma in type 2 diabetes mellitus
hypoglycaemic coma in type ii diabetes mellitus
hypoglycemic coma in type 2 diabetes mellitus
hypoglycemic coma in type ii diabetes mellitus
insulin dependent diabetes mellitus with hypoglycaemic coma
insulin dependent diabetes mellitus with hypoglycemic coma
ketoacidotic coma in insulin-dependent diabetes mellitus
ketoacidotic coma in juvenile-onset type diabetes mellitus
ketoacidotic coma in type 1 diabetes mellitus
ketoacidotic coma in type i diabetes mellitus
type 1 diabetes mellitus with hypoglycaemic coma
type 1 diabetes mellitus with hypoglycemic coma
type i diabetes mellitus with hypoglycaemic coma
type i diabetes mellitus with hypoglycemic coma
diabetic renal disease
renal disorder associated with diabetes mellitus
kidney disorder associated with juvenile-onset type diabetes mellitus
kidney disorder associated with type 1 diabetes mellitus
renal disorder associated with insulin dependent diabetes mellitus
renal disorder associated with type 1 diabetes mellitus
renal disorder associated with type i diabetes mellitus
advanced diabetic maculopathy
advanced diabetic retinal disease

background diabetic retinopathy
bdr background diabetic retinopathy
diabetic cataract associated with type 2 diabetes mellitus
diabetic cataract associated with type ii diabetes mellitus
diabetic disc edema
diabetic disc oedema
diabetic eye disease
diabetic intraretinal microvascular anomalies
diabetic intraretinal microvascular anomaly
diabetic macular edema
diabetic macular edema not clinically significant
diabetic macular oedema
diabetic macular oedema not clinically significant
diabetic oculopathy associated with type 2 diabetes mellitus
diabetic oculopathy associated with type ii diabetes mellitus
diabetic optic papillopathy
diabetic retinal microaneurysm
diabetic retinal venous beading
diabetic retinopathy 12 month review
diabetic retinopathy 6 month review
diabetic retinopathy associated with type 2 diabetes mellitus
diabetic retinopathy associated with type ii diabetes mellitus
diabetic traction retinal detachment
diabetic vitreous haemorrhage
diabetic vitreous haemorrhage associated with type 2 diabetes mellitus
diabetic vitreous haemorrhage associated with type ii diabetes mellitus
diabetic vitreous hemorrhage
diabetic vitreous hemorrhage associated with type 2 diabetes mellitus
diabetic vitreous hemorrhage associated with type ii diabetes mellitus
diffuse diabetic maculopathy
dr diabetic retinopathy
early proliferative diabetic retinopathy
exudative maculopathy associated with type 1 diabetes mellitus
exudative maculopathy associated with type 2 diabetes mellitus
exudative maculopathy associated with type i diabetes mellitus
exudative maculopathy associated with type ii diabetes mellitus
focal diabetic maculopathy
high risk pdr
high risk pdr not amenable to photcoagulation
high risk pdr not amenable to photocoagulation
high risk proliferative diabetic retinopathy
high risk proliferative diabetic retinopathy not amenable to photocoagulation
high risk proliferative retinopathy
inactive proliferative diabetic retinopathy
intraretinal microvascular abnormality
intraretinal microvascular anomalies
intraretinal microvascular anomaly
irma intraretinal microvascular anomaly
ischaemic diabetic maculopathy
ischemic diabetic maculopathy
mixed diabetic maculopathy
non proliferative diabetic retinopathy
non-high risk pdr
non-high risk pdr with csme
non-high-risk pdr with no macular edema
non-high-risk pdr with no macular oedema
non-high-risk proliferative diabetic retinopathy with clinically significant macular edema
non-high-risk proliferative diabetic retinopathy with clinically significant macular oedema
non-high-risk proliferative diabetic retinopathy with no macular edema
non-high-risk proliferative diabetic retinopathy with no macular oedema
nonproliferative diabetic retinopathy
nonproliferative diabetic retinopathy associated with type 2 diabetes mellitus
nonproliferative diabetic retinopathy associated with type ii diabetes mellitus
npdr non proliferative diabetic retinopathy
o/e diabetic maculopathy present both eyes
o/e left eye background diabetic retinopathy
o/e left eye diabetic maculopathy
o/e left eye preproliferative diabetic retinopathy
o/e left eye proliferative diabetic retinopathy
o/e left eye stable treated proliferative diabetic retinopathy

o/e right eye background diabetic retinopathy
o/e right eye diabetic maculopathy
o/e right eye proliferative diabetic retinopathy
o/e right eye stable treated proliferative diabetic retinopathy
o/e sight threatening diabetic retinopathy
on examination diabetic maculopathy present both eyes
on examination left eye background diabetic retinopathy
on examination left eye diabetic maculopathy
on examination left eye preproliferative diabetic retinopathy
on examination left eye proliferative diabetic retinopathy
on examination left eye stable treated proliferative diabetic retinopathy
on examination right eye background diabetic retinopathy
on examination right eye diabetic maculopathy
on examination right eye proliferative diabetic retinopathy
on examination right eye stable treated proliferative diabetic retinopathy
on examination sight threatening diabetic retinopathy
ophthalmic complication of adult-onset type diabetes mellitus
ophthalmic complication of malnutrition-related diabetes mellitus
ophthalmic complication of non-insulin-dependent diabetes mellitus
ophthalmic manifestations of diabetes
pdr high risk with csme
pdr high risk with no macular edema
pdr high risk with no macular oedema
pdr iris neovascularisation
pdr iris neovascularization
pdr new vessels on disc
pdr non-high risk
pdr proliferative diabetic retinopathy
ppdr preproliferative diabetic retinopathy
preproliferative diabetic retinopathy
proliferative diabetic retinopathy
proliferative diabetic retinopathy associated with type 2 diabetes mellitus
proliferative diabetic retinopathy associated with type ii diabetes mellitus
proliferative diabetic retinopathy high risk
proliferative diabetic retinopathy high risk with clinically significant macular edema
proliferative diabetic retinopathy high risk with clinically significant macular oedema
proliferative diabetic retinopathy high risk with no macular edema
proliferative diabetic retinopathy high risk with no macular oedema
proliferative diabetic retinopathy iris neovascularisation
proliferative diabetic retinopathy iris neovascularization
proliferative diabetic retinopathy new vessels on disc
proliferative diabetic retinopathy non high risk
proliferative diabetic retinopathy quiescent
proliferative diabetic retinopathy with new vessels elsewhere than on disc
proliferative diabetic retinopathy with new vessels on disc
proliferative retinopathy new vessels on disc
proliferative retinopathy with new vessels elsewhere than on disc
proliferative retinopathy with new vessels elswhere than on disc
retinal abnormality diabetes-related
retinal venous beading
retinopathy associated with adult-onset type diabetes mellitus
retinopathy associated with non-insulin-dependent diabetes mellitus
severe non proliferative diabetic retinopathy
severe nonproliferative diabetic retinopathy
severe nonproliferative diabetic retinopathy with clinically significant macular edema
severe nonproliferative diabetic retinopathy with clinically significant macular oedema
severe nonproliferative diabetic retinopathy with no macular edema
severe nonproliferative diabetic retinopathy with no macular oedema
severe nonproliferative retinopathy
severe npdr with csme
severe npdr with no macular edema
severe npdr with no macular oedema
sight threatening diabetic retinopathy
very severe nonproliferative diabetic retinopathy
very severe nonproliferative diabetic retinopathy with clinically significant macular edema
very severe nonproliferative diabetic retinopathy with clinically significant macular oedema
very severe nonproliferative diabetic retinopathy with no macular edema
very severe nonproliferative diabetic retinopathy with no macular oedema
very severe npdr
very severe npdr with csme

very severe npdr with no macular edema
very severe npdr with no macular oedema
very severe proliferative diabetic retinopathy
visually threatening diabetic retinopathy
diabetic oculopathy associated with type 1 diabetes mellitus
diabetic oculopathy associated with type i diabetes mellitus
acute painful diabetic neuropathy
amyotrophy due to type 2 diabetes mellitus
asymmetric diabetic proximal motor neuropathy
asymptomatic diabetic neuropathy
autonomic neuropathy due to diabetes
chronic painful diabetic neuropathy
diabetes mellitus with neuropathy
diabetic acute painful polyneuropathy
diabetic asymmetric polyneuropathy
diabetic autonomic neuropathy
diabetic autonomic neuropathy associated with type 1 diabetes mellitus
diabetic autonomic neuropathy associated with type 2 diabetes mellitus
diabetic charcot's arthropathy
diabetic charcot's arthropathy associated with type 2 diabetes mellitus
diabetic chronic painful polyneuropathy
diabetic distal sensorimotor polyneuropathy
diabetic femoral mononeuropathy
diabetic gastroparesis associated with type 1 diabetes mellitus
diabetic gastroparesis associated with type 2 diabetes mellitus
diabetic mixed sensory-motor polyneuropathy
diabetic mononeuritis multiplex
diabetic mononeuropathy multiplex
diabetic mononeuropathy simplex
diabetic motor polyneuropathy
diabetic neurologic disease
diabetic neuropathic arthropathy
diabetic neuropathic arthropathy associated with type 2 diabetes mellitus
diabetic neuropathy with neurologic complication
diabetic peripheral neuropathy
diabetic peripheral neuropathy associated with type 2 diabetes mellitus
diabetic peripheral neuropathy associated with type ii diabetes mellitus
diabetic sensory polyneuropathy
diabetic thoracic radiculopathy
diabetic truncal radiculopathy
mononeuropathy associated with non-insulin dependent diabetes mellitus
mononeuropathy associated with type 2 diabetes mellitus
mononeuropathy associated with type ii diabetes mellitus
myasthenic syndrome due to diabetic amyotrophy
neurologic complication of adult-onset type diabetes mellitus
neurologic complication of diabetes mellitus
neurologic disorder associated with diabetes mellitus
neurologic disorder associated with type 2 diabetes mellitus
neurologic disorder associated with type ii diabetes mellitus
neurological disorder associated with malnutrition-related diabetes mellitus
non-insulin dependent diabetes mellitus with neuropathic arthropathy
polyneuropathy associated with adult-onset type diabetes mellitus
polyneuropathy associated with juvenile-onset diabetes mellitus
polyneuropathy associated with non-insulin dependent diabetes mellitus
polyneuropathy associated with type 1 diabetes mellitus
polyneuropathy associated with type 2 diabetes mellitus
polyneuropathy associated with type i diabetes mellitus
polyneuropathy associated with type ii diabetes mellitus
symmetric diabetic proximal motor neuropathy
type 1 diabetic autonomic neuropathy
type 2 diabetes mellitus with neuropathic arthropathy
type ii diabetes mellitus with neuropathic arthropathy
neurologic complication of juvenile-onset diabetes mellitus
neurological disorder associated with type 1 diabetes mellitus
neurological disorder associated with type i diabetes mellitus
diabetic peripheral circulatory disorder
peripheral circulatory disorder associated with diabetes mellitus
insulin-dependent diabetes mellitus with gangrene
type 1 diabetes mellitus with gangrene
type i diabetes mellitus with gangrene

angina associated with type 2 diabetes mellitus
angina associated with type ii diabetes mellitus
ballinger-wallace syndrome
cellulitis in diabetic foot
diabetes insipidus,diabetes mellitus optic atrophy and deafness
diabetes mellitus and insipidus with optic atrophy and deafness
diabetes mellitus associated with cystic fibrosis
diabetes mellitus associated with genetic syndrome
diabetes mellitus associated with hormonal aetiology
diabetes mellitus associated with hormonal etiology
diabetes mellitus due to cystic fibrosis
diabetes mellitus due to insulin receptor antibodies
diabetes-deafness syndrome maternally transmitted
diabetic angina pectoris associated with type 2 diabetes mellitus
diabetic dermopathy associated with diabetes mellitus type 2
diabetic dyslipidemia associated with type 2 diabetes mellitus
diabetic foot ulcer
diabetic foot ulcer associated with type 2 diabetes mellitus
diabetic foot ulcer associated with type ii diabetes mellitus
diabetic hand syndrome
diabetic necrobiosis lipoidica
diabetic skin ulcer
diabetic skin ulcer associated with type 2 diabetes mellitus
diabetic thick skin syndrome
diarrhea in diabetes
diarrhoea in diabetes
didmoad diabetes insipidus,diabetes mellitus optic atrophy and deafness
dm due to insulin receptor ab
dyslipidemia associated with type ii diabetes mellitus
erectile dysfunction associated with type 2 diabetes mellitus
fibrocalculous pancreatic diabetes
genetic syndromes of diabetes mellitus
hyperglycaemic crisis in diabetes mellitus
hyperglycemic crisis in diabetes mellitus
hypoglycaemic event in diabetes
hypoglycaemic state in diabetes
hypoglycemic event in diabetes
hypoglycemic state in diabetes
infection of foot associated with diabetes
insulin-resistant diabetes mellitus and acanthosis nigricans
ischaemic ulcer diabetic foot
ischemic ulcer diabetic foot
loss of hypoglycaemic warning
loss of hypoglycemic warning
malnutrition-related diabetes mellitus fibrocalculous
marquardt-loriaux syndrome
mixed diabetic ulcer foot
mixed hyperlipidaemia associated with type 2 diabetes mellitus
mixed hyperlipidaemia associated with type ii diabetes mellitus
mixed hyperlipidemia associated with type 2 diabetes mellitus
mixed hyperlipidemia associated with type ii diabetes mellitus
necrobiosis lipoidica diabeticorum
neuropathic diabetic ulcer foot
nld necrobiosis lipoidica diabeticorum
non-insulin-dependent diabetes mellitus with ulcer
o/e left chronic diabetic foot ulcer
o/e left diabetic foot at high risk
o/e left diabetic foot at low risk
o/e left diabetic foot at moderate risk
o/e left diabetic foot at risk
o/e left diabetic foot ulcerated
o/e right chronic diabetic foot ulcer
o/e right diabetic foot at high risk
o/e right diabetic foot at low risk
o/e right diabetic foot at moderate risk
o/e right diabetic foot at risk
o/e right diabetic foot ulcerated
on examination left chronic diabetic foot ulcer
on examination left diabetic foot at high risk
on examination left diabetic foot at low risk

on examination left diabetic foot at moderate risk
on examination left diabetic foot at risk
on examination left diabetic foot ulcerated
on examination right chronic diabetic foot ulcer
on examination right diabetic foot at high risk
on examination right diabetic foot at low risk
on examination right diabetic foot at moderate risk
on examination right diabetic foot at risk
on examination right diabetic foot ulcerated
pretibial pigmental patches in diabetes
skin ulcer associated with diabetes mellitus
skin ulcer associated with type ii diabetes mellitus
small vessel disease due to type 2 diabetes mellitus
soft tissue complication of diabetes mellitus
type 2 diabetes mellitus with acanthosis nigricans
type 2 diabetes mellitus with ulcer
type ii diabetes mellitus with ulcer
congenital insulin-dependent diabetes mellitus with fatal secretory diarrhea
congenital insulin-dependent diabetes mellitus with fatal secretory diarrhoea
diabetic erectile dysfunction associated with type 1 diabetes mellitus
erectile dysfunction associated with type 1 diabetes mellitus
erectile dysfunction associated with type i diabetes mellitus
iddm insulin-dependent diabetes mellitus secretory diarrhea syndrome
iddm insulin-dependent diabetes mellitus secretory diarrhoea syndrome
insulin-dependent diabetes mellitus secretory diarrhea syndrome
insulin-dependent diabetes mellitus secretory diarrhoea syndrome
insulin-dependent diabetes mellitus with ulcer
mixed hyperlipidaemia associated with type 1 diabetes mellitus
mixed hyperlipidemia associated with type 1 diabetes mellitus
small vessel disease due to type 1 diabetes mellitus
type 1 diabetes mellitus with ulcer
type i diabetes mellitus with ulcer
complication of adult-onset type diabetes mellitus
complication of type ii diabetes mellitus
disorder associated with type 2 diabetes mellitus
disorder associated with type ii diabetes mellilltus
disorder associated with type ii diabetes mellitus
complication of insulin-dependent diabetes mellitus
complication of juvenile-onset type diabetes mellitus
complication of type i diabetes mellitus
disorder associated with type 1 diabetes mellitus
disorder associated with type i diabetes mellitus
glycosuria
ketonuria
