# Supplementary material for: Web-based Real-Time Case Finding for the Population Health Management of Patients With Diabetes Mellitus: A Prospective Validation of the Natural Language Processing–Based Algorithm With Statewide Electronic Medical Records
Source: JMIR Med Inform. 2016 Nov 11;4(4):e37. doi: 10.2196/medinform.6328 (PMC5124114; doi:10.2196/medinform.6328)
Supplement: Multimedia Appendix 2 [file medinform_v4i4e37_app2.pdf]

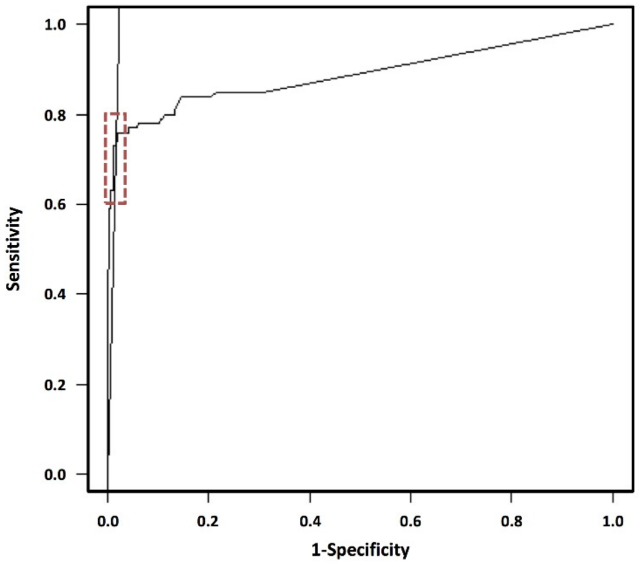

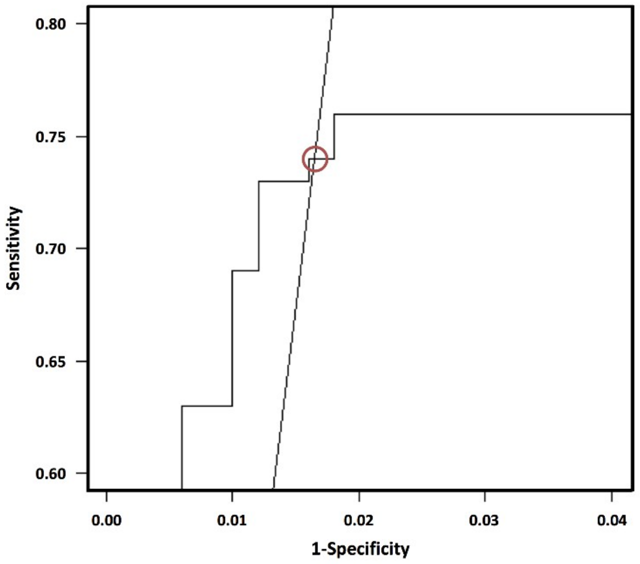

Multimedia Appendix 1. Determination of the cutoff point of the subject classification probabilities. Top: The ROC curve, and the line determined by the prevalence and 90% PPV,

were intersected at the cutoff point. Bottom: Their intersection (dashed rectangle) was zoomed up and indicated by the circle.
