# Supplementary material for: Web-based Real-Time Case Finding for the Population Health Management of Patients With Diabetes Mellitus: A Prospective Validation of the Natural Language Processing–Based Algorithm With Statewide Electronic Medical Records
Source: JMIR Med Inform. 2016 Nov 11;4(4):e37. doi: 10.2196/medinform.6328 (PMC5124114; doi:10.2196/medinform.6328)
Supplement: Multimedia Appendix 3 [file medinform_v4i4e37_app3.pdf]

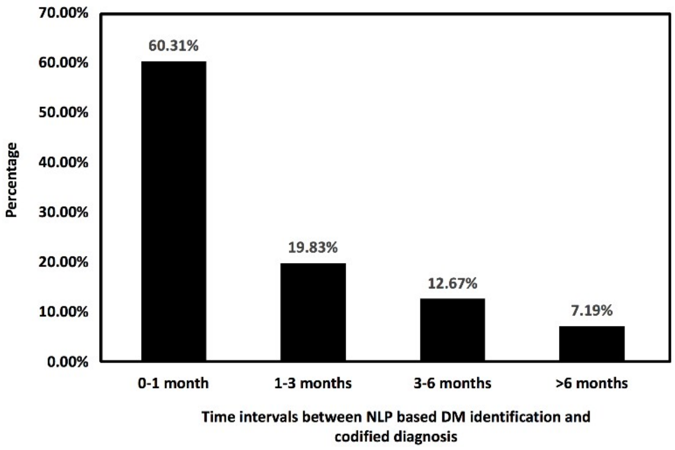

Multimedia Appendix 2. The distribution of patients by the time intervals between NLP based DM identification and codified diagnosis.
